# Pharmaceutical payments to Japanese board-certified dermatologists: a 4-year retrospective analysis of personal payments from pharmaceutical companies between 2016 and 2019

**DOI:** 10.1038/s41598-023-34705-8

**Published:** 2023-05-08

**Authors:** Anju Murayama, Sae Kamamoto, Hiroaki Saito, Akihiko Ozaki

**Affiliations:** 1 Tohoku University School of Medicine, Sendai, Miyagi Japan; 2 Faculty of Medicine, Hamamatsu University School of Medicine, Hamamatsu, Shizuoka Japan; 3 Medical Governance Research Institute, Minato-Ku, Tokyo, Japan; 4 Department of Internal Medicine, Soma Central Hospital, Soma, Fukushima Japan; 5 Department of Breast Surgery, Jyoban Hospital of Tokiwa Foundation, Iwaki, Fukushima Japan

**Keywords:** Medical research, Epidemiology, Health care economics, Health policy, Health services, Medical ethics

## Abstract

There are prevalent financial relationships between dermatologists and pharmaceutical companies in Japan. However, little was known about the extent of whole picture of the personal payments made to dermatologists by pharmaceutical companies. This study aimed to examine the personal payments to the board-certified dermatologists by the Japanese Dermatological Association from the pharmaceutical companies between 2016 and 2019. Using the publicly disclosed payments data by the pharmaceutical companies between 2016 and 2019, we evaluated the magnitude, prevalence, and trends in the personal payments made to all board-certified dermatologists for the lecturing, writing, and consulting compensations. The payments were descriptively analyzed overall and by dermatologist demographics. Additionally, the payment trends were assessed by generalized estimating equation models. Of 6883 active board-certified dermatologists, 3121 (45.3%) received a total of $33,223,806 personal payments between 2016 and 2019. The median per-physician payments and number of payments (interquartile range) were $1737 ($613–$5287) and 4.0 (2.0–10.0) over the 4 years, respectively. Only top 1%, 5%, 10% of dermatologists received 41.7% (95% confidence interval [CI] 38.2–45.1%), 76.9% (95% CI 74.7–79.1%), and 87.6% (95% CI 86.2–88.9%) of overall payments. The number of dermatologists receiving payments and per-dermatologist payments increased by 4.3% (95% CI 3.1‒5.5%, p < 0.001) and 16.4% (95% CI 13.5‒19.4%, p < 0.001) each year. The board-certification in dermatology-oncology, in cosmetic dermatology, and male sex were significantly associated with higher personal payments with relative monetary values of 2.29 (95% CI 1.65–3.19, p < 0.001), 3.16 (95% CI 1.89–5.26, p < 0.001), and 5.38 (95% CI 4.12–7.04, p < 0.001). Less than half of Japanese board-certified dermatologists received lower personal payments from the pharmaceutical companies than those to other specialists. However, these personal payments were increasingly more prevalent and greater over the 4 years.

## Introduction

Financial collaboration between physicians and pharmaceutical companies can benefit patients by deepening understanding of illnesses and developing their new diagnostic methods and treatments, but also harm patient care by expensive and inappropriate prescriptions, biasing guideline recommendations favorable for pharmaceutical industry, and jeopardizing results and interpretations of clinical trial. In response to public call for greater transparency in the physician-industry financial relationships, transparency acts and guidelines were developed and the financial transfers made by pharmaceutical companies to physicians were publicly disclosed in many developed countries, such as the Physician Payment Sunshine Act and the Open Payments Database in the United States^1,2^. In Japan, instead of legislative regulation, Japan Pharmaceutical Manufacturers Association (JPMA), the largest trade organizations of major pharmaceutical companies in Japan, published a transparency guidance and the payments for lecturing, consulting, and writing compensations to physicians have been voluntarily disclosed by each pharmaceutical company affiliated to the JPMA since 2013^3^.

Due to this payment disclosure, we previously reported there were substantial and prevalent financial relationships between pharmaceutical companies and physicians in several specialties including oncology, hematology, pediatrics, infectious diseases, and pulmonology in Japan^4–8^. These personal payments were often made to physicians in authoritative and influential positions such as clinical practice guideline authors^6,9–12^, society board members^6,13^, university professors^6,7,14^, and television expert commentators^15^. We also found that there was a pattern in recipient of personal payments by factors such as gender and regions.

Among several specialties, dermatologists have had strong financial ties to the pharmaceutical companies in Japan. Indeed, executive board members of the Japanese Dermatological Association (JDA) received the second highest payments in the median amounts among those representing 18 major clinical medicine specialties in Japan^13^. Additionally, 90.6% of the JDA clinical practice guideline authors received $10,281 in median personal payments between 2016 and 2017^16^. In 13 out of 32 (40.6%) JDA guidelines, all authors accepted personal payments from the pharmaceutical companies^16^. These payments were disproportionately distributed to physicians by the demographic differences including gender^16^.

Additionally, a previous study assessing patients’ awareness and perception of financial relationships between physicians and pharmaceutical companies demonstrated that the majority of patients expect physicians to be transparent about their financial conflicts of interest with pharmaceutical companies and to minimize the non-research financial relationships such as speaking fees, gifts and meals from pharmaceutical companies in Japan^17^. Growing body of evidence mostly from the United States and other developed countries show that the non-research payments to physicians significantly influence physicians’ prescribing patterns leading to increased prescriptions and healthcare costs^18–25^. Reducing these non-research payments to physicians might lead to more proper and balanced care, as well as greater transparency and independence of physicians. Thus, as a first step towards the above goal, the investigation of financial relationships between physicians and pharmaceutical companies is of particular importance for all patients, healthcare professionals, and policymakers.

Nevertheless, there was no document describing the whole magnitude and prevalence of dermatologists accepting personal payments from the pharmaceutical companies in Japan. Considering that 8333, equal to 73.7% of all dermatologists, received more than $34 million in the United States in 2014^26^, we hypothesized that there were substantial and prevalent financial relationships between dermatologists and pharmaceutical companies in Japan. This study purposed to evaluate magnitude, prevalence, and trends in personal payments from pharmaceutical companies to dermatologists in Japan for the recent years.

## Methods

### Study design and participants

This cross-sectional analysis examined the personal payments for the lecturing, consulting, and writing proposes made to board-certified dermatologists by the JDA from the JPMA member companies between 2016 and 2019. The board certification in dermatology was one of the nineteen basic specialties in Japan.

This study included all dermatologists board-certified by the JDA as of September 30, 2021. The JDA was established in 1900 and the largest and sole professional medical association certifying dermatologists in Japan. Names, affiliations, JDA-certified specialist certification in dermatology-oncology or cosmetic dermatology, and gender of all board-certified dermatologists were publicly available from the JDA webpage (https://www.dermatol.or.jp/modules/spMap/doctors?pref=&sp=1&words =).

### Data collection

The payment data considering lecturing, consulting, and writing compensations were disclosed at individual level by the pharmaceutical companies. Definitions of payment categories were described previously^5,6^. We collected payment data from all 92 pharmaceutical companies belonging to the JPMA to the board-certified dermatologists between 2016 and 2019, sorting by dermatologists’ names, as described previously^7,12,16^. The payments in 2019 were the latest analyzable payment data as of August 2022. The payment recipients’ affiliations were matched to the JDA-reported dermatologists’ affiliation, in order to delete payment data to different person with duplicate name^27^. As for a payment which we could not verify, we excluded the payment from the analysis. The detailed procedure was noted previously^5,11,28^. Information on whether dermatologists work in facilities accredited by the JDA for the use of biologics was collected from the JDA webpage.

### Analysis

We performed descriptive analysis on the payment data including average (standard deviation [SD]) and median (interquartile range [IQR]) payments and number of payments. Per-physician payments were calculated based on dermatologists receiving payments, as in other studies^2,26,29^. Payment concentration were evaluated by the Gini index and the shares of the value of payments held by the top 1%, 5%, 10%, and 25% of dermatologists. The Gini index ranges from 0 to 1, and the greater the Gini index, the greater the disparity in the distribution of payments on the specialist basis^11^. Additionally, the trends in annual personal payments to dermatologists were examined by population-averaged generalized estimating equation (GEE) models with panel-data of payments clustering each dermatologist between 2016 and 2019^5,6^. The log-linked linear GEE model with Poisson distribution for the number of dermatologists receiving payments and negative binomial regression GEE model for the per-dermatologist payments were applied, as the payments were highly skewed. As several pharmaceutical companies disaffiliated from or newly affiliated to the JPMA between 2016 and 2019, the payment trends in industry payments were calculated based on the payments from the companies continuously affiliated with JPMA throughout the 4 years. Separately, we examined the associations between (1) the 4-year total payment amounts and (2) the likelihood of dermatologists to receive payments and the dermatologist demographic characteristics using (1) a multivariable negative binomial model and (2) a modified log-linked Poisson regression model, respectively. Gender, whether a dermatologist had board-certification in dermatology-oncology or cosmetic dermatology, practicing regions, and whether a dermatologist works in facilities accredited by the JDA for the use of biologics were set as independent variables, and the number of dermatologists receiving payments and per-dermatologist payment values were set as dependent variables, respectively^29–31^. Japanese yen (¥) was converted into dollars ($) using 2019 average monthly exchange rate of ¥109.0 per $1, respectively. All analyses were conducted using Microsoft Excel, version 16.0 (Microsoft Corp) and Stata version 17.0 (StataCorp).

### Ethical approval

The Ethics Committee of the Medical Governance Research Institute approved this study. This study is a retrospective cross-sectional analysis of publicly available information so informed consent from participants were waived by the Ethics Committee of the Medical Governance Research Institute. This study was performed in accordance with relevant guidelines/regulations.

## Results

There were 6883 board-certified dermatologists by JDA including 3556 (51.7%) male, 92 (1.3%) dermatology-oncology specialists, and 49 (0.7%) cosmetic dermatology specialists. Of 6883 board-certified dermatologists by JDA, 3121 (45.3%) received 43,475 personal payments totaling $33,223,806 between 2016 and 2019. The median per-physician payments, number of payments, and number of companies making payments were $1737 (IQR $613–$5287), 4.0 (IQR 2.0–10.0), and 3.0 (IQR 1.0–6.0) over the 4 years, respectively (Table 1) The number of dermatologists receiving more than $10,000, $50,000, and $100,000 in the 4-year combined total were 511 (7.4%), 159 (2.3%), and 72 (1.1%), respectively. Only top 1%, 5%, 10% of dermatologists received 41.7%, 76.9%, and 87.6% of overall payments. Gini index for per-physician 4-year total payments was 0.917, indicating that only a small number of dermatologists received substantial amounts of personal payments from the pharmaceutical companies. Of 3121 dermatologists with payments, 68.3% (2133 dermatologists) received payments from more than one company.

**Table 1:** Summary of personal payments from pharmaceutical companies to board-certified dermatologists between 2016 and 2019.

Variables	Values
Total	
Payment values, $	33,223,806
Cases, n	43,475
Companies, n	76
Average per dermatologist (standard deviation)	
Payment values, $	10,645 (34,740)
Cases, n	13.9 (33.7)
Companies, n	4.4 (4.7)
Median per dermatologist (interquartile range)	
Payment values, $	1737 (613‒5287)
Cases, n	4.0 (2.0‒10.0)
Companies, n	3.0 (1.0‒6.0)
Range	
Payment values, $	94‒495,550
Cases, n	1.0‒490.0
Companies, n	1.0‒34.0
Physicians with specific payments, n (%)	
Any payments	3121 (45.3)
Payments > $500	2580 (37.5)
Payments > $1000	2003 (29.1)
Payments > $5000	812 (11.8)
Payments > $10,000	511 (7.4)
Payments > $50,000	159 (2.3)
Payments > $100,000	72 (1.1)
Gini index	0.917

As for payment categories, lecturing payments occupied 81.2% in monetary amounts and 84.5% of all payments in the number of payments. (Table 2) Consulting payments were the highest per-payment value with an average of $1009, while the lecturing payment was the lowest per-payment value with $735. Among 6883 dermatologists, 2954 (42.9%), 1210 (17.6%), and 533 (8.0%) received one or more payments for lecturing, consulting, and writing compensations from the pharmaceutical companies over the 4 years.

**Table 2:** Payments by categories.

Category of payments	Payment amounts, $ (%)	Number of payments, n (%)	Number of dermatologists, n (%)
Lecturing	26,993,658 (81.2)	36,729 (84.5)	2954 (42.9)
Consulting	4,335,831 (13.1)	4294 (9.9)	1210 (17.6)
Writing	1,852,087 (5.6)	2406 (5.5)	553 (8.0)
Other	42,230 (0.1)	46 (0.1)	31 (0.5)

Table 3 shows the annual trends in personal payments to the dermatologists between 2016 and 2019. The total personal payments increased from $6,507,920 in 2016 to $9,536,625 in 2018, and $9,422,603 in 2019. The number of dermatologists receiving payments also increased by 4.3% (95% CI 3.1‒5.5%, p < 0.001) annually, from 1800 (26.2%) in 2016 to 2042 (29.7%) in 2018. Median annual personal payments per dermatologist were the highest in 2018, with $1046 (IQR $511‒$3024). Per-dermatologist payments significantly increased by 14.1% (95% CI 11.2‒16.9%, p < 0.001) each year.

**Table 3:** Trends in personal payments from pharmaceutical companies to board-certified dermatologists between 2016 and 2019.

Variables	2016	2017	2018	2019	Relative average annual percentage change (95% confidence interval), %	P value	Combined total
Total payments, $	6,507,920	7,756,658	9,536,625	9,422,603	‒	‒	33,223,806
Average payments (standard deviation), $	3616 (10,398)	4042 (10,734)	4670 (11,889)	4635 (11,467)	14.1 (11.2 to 16.9)	< 0.001	10,645 (34,740)
Median payments (interquartile range), $	882 (473‒2229)	984 (511‒2399)	1046 (511‒3024)	1031 (511‒2932)			1737 (613‒5287)
Payment range, $	51‒162,327	51‒137,625	51‒155,719	82‒137,319	‒	‒	94‒495,550
Physicians with specific payments, n (%)							
Any payments	1,800 (26.2)	1,919 (27.9)	2,042 (29.7)	2,033 (29.5)	4.3 (3.1 to 5.5)	< 0.001	3,121 (45.3)
Payments > $500	1,328 (19.3)	1,488 (21.6)	1,603 (23.3)	1,599 (23.2)	6.2 (4.8 to 7.7)	< 0.001	2,580 (37.5)
Payments > $1,000	824 (12.0)	944 (13.7)	1085 (15.8)	1092 (15.9)	10.0 (8.2 to 11.8)	< 0.001	2,003 (29.1)
Payments > $5,000	238 (3.5)	273 (4.0)	345 (5.0)	353 (5.1)	14.7 (11.4 to 18.2)	< 0.001	812 (11.8)
Payments > $10,000	139 (2.0)	166 (2.4)	205 (3.0)	221 (3.2)	16.8 (12.3 to 21.5)	< 0.001	511 (7.4)
Payments > $50,000	19 (0.3)	25 (0.4)	33 (0.5)	30 (0.4)	15.2 (2.9 to 28.9)	0.014	159 (2.3)
Payments > $100,000	3 (0.0)	4 (0.1)	5 (0.1)	4 (0.1)	8.3 (− 29.6 to 66.8)	0.72	72 (1.1)
Gini index	0.935	0.931	0.926	0.926	‒	‒	0.917

Additionally, among 3556 male dermatologists, 2087 (58.7%) accepted more than one personal payments from the pharmaceutical companies over 4 years (Table 4). Meanwhile, 1034, equal to 31.1% of female dermatologists, received personal payments. Male dermatologists were more likely to accept personal payments from the pharmaceutical companies over the 4 years (p < 0.001 in Chi-square test). Median per-dermatologist payments were $2414 (IQR $817–$7480) in male and $984 (IQR $473–$2554) in female. The male dermatologists were 1.88 (95% CI 1.78–1.99, p < 0.001) times more likely to accept personal payments and received 4.63 (95% CI 3.57–6.02, p < 0.001) times larger personal payments per dermatologist than female.

**Table 4:** Payments by physician demographic characteristics.

Variables	Number of dermatologists, n (%)	Dermatologists accepting payments, n (%)	Relative likelihood to accept payments (95% confidence interval), times	Total payments, $	Median per-dermatologist payments (interquartile range), $	Average per-dermatologist payments (standard deviation), $	Relative monetary value (95% confidence interval), times
Gender							
Female	3327 (48.3)	1034 (31.1)	Ref	5,125,097	984 (473–2,554)	4957 (20,910)	Ref
Male	3556 (51.7)	2087 (58.7)	1.88 (1.78–1.99)***	28,098,709	2414 (817–7,480)	13,464 (39,555)	4.63 (3.57–6.02)***
Subspecialty							
General dermatology	6742 (98.0)	2990 (44.4)	Ref	31,067,581	1643 (590–4,985)	10,390 (35,033)	Ref
Oncology dermatology	92 (1.3)	89 (96.7)	1.37 (1.28–1.47)***	1,524,332	6464 (2,059–17,793)	17,127 (26,067)	2.73 (1.63–4.60)***
Cosmetic dermatology	49 (0.7)	42 (85.7)	1.74 (1.53–1.97)***	631,893	3365 (1,505–12,475)	15,045 (28,159)	3.91 (1.92–7.97)***
Region							
Kanto	2619 (38.1)	1084 (41.4)	Ref	13,406,309	1675 (613–5,332)	12,367 (42,583)	Ref
Hokkaido	280 (4.1)	129 (46.1)	0.98 (0.87–1.11)	1,695,222	2683 (946–6,186)	13,141 (37,898)	0.71 (0.46–1.08)
Tohoku	364 (5.3)	199 (54.7)	1.17 (1.06–1.29)**	1,815,440	2099 (882–5,848)	9123 (26,150)	0.84 (0.57–1.23)
Chubu	1003 (14.6)	454 (45.3)	1.02 (0.95–1.10)	4,722,211	1679 (613–5,652)	10,401 (31,193)	0.75 (0.49–1.15)
Kinki	1260 (18.3)	585 (46.4)	1.08 (1.00–1.15)	5,826,077	1726 (590–5,539)	9959 (28,827)	0.71 (0.50–0.99)*
Chugoku	389 (5.7)	200 (51.4)	1.19 (1.08–1.31)**	1,900,130	1915 (715–4,640)	9501 (31,129)	1.22 (0.60–2.48)
Shikoku	199 (2.9)	98 (49.3)	1.11 (0.96–1.28)	820,153	1981 (903–5,941)	8369 (20,818)	0.84 (0.49–1.46)
Kyusyu	723 (10.5)	353 (48.8)	1.09 (1.00–1.18)*	3,015,849	1378 (517–4,092)	8543 (29,802)	0.74 (0.50–1.11)
Abroad and unknown	46 (0.7)	19 (41.3)	1.18 (0.85–1.63)	22,415	914 (307–1,230)	1180 (1,394)	0.28 (0.14–0.54)***
JDA-accredited facility for biologics use							
No	4773 (69.3)	1809 (37.9)	Ref	7,702,058	1230 (511–3241)	4258 (16,762)	Ref
Yes	2110 (30.7)	1312 (62.2)	1.65 (1.57–1.73)***	25,521,748	3118 (958–12,898)	19,453 (48,485)	6.99 (5.47–8.93)***

*p < 0.05, **p < 0.01, ***p < 0.001.

Of 92 dermatology-oncology specialists and 49 cosmetic dermatology specialists, 89 (96.7%) and 42 (85.7%) received personal payments, while 44.3% (2990 out of 6742) of JDA board-certified dermatologists did. (Table 4) Median per-dermatologists payments were $1643 (IQR: $590–$4985) in board-certified dermatologists, $6464 (IQR: $2059–$17,793) in board-certified dermatology-oncology specialists, and $3365 (IQR: $1505–$12,475) in board-certified cosmetic dermatology specialists. The board-certified dermatology-oncology specialists and board-certified cosmetic dermatology specialists were 1.37 (95% CI 1.28–1.47, p < 0.001) times and 1.74 (95% CI 1.53–1.97, p < 0.001) times more likely to receive payments than the board-certified dermatologists. The per-dermatologist payments were 2.73 (95% CI 1.63–4.60, p < 0.001) times in board-certified dermatology-oncology specialists and 3.91 (95% CI 1.92–7.97, p < 0.001) times in board-certified cosmetic dermatology specialists larger than those in the board-certified dermatologists. Not surprisingly, the dermatologists working in a facility accredited by the JDA for the use of biologics were 1.65 times (95% CI 1.57–1.73, p < 0.001) more likely to receive personal payments and per-dermatologist payment values were 6.99 times (95% CI 5.47–8.93, p < 0.001) higher in dermatologists working in JDA-accredited facilities than those without.

Table 5 showed the lists of ten pharmaceutical companies making the largest personal payments between 2016 and 2019. Of 75 companies making payments, payments for ten companies with top-largest payments accounted for 73.4% of overall payment amounts. Maruho made the highest number of payments and largest total payments to the highest number of dermatologists, with 6888 (15.8% of overall number of payments) payments totaling $6,003,214 (18.1% of overall payment amounts) to 1515 equal to 22.0% of all dermatologists over the 4 years. Of the top ten companies, five including Taiho Pharmaceutical, Novartis, Sanofi, Eli Lilly, and Eisai made increasingly personal payments to the dermatologists between 2016 and 2019. The total payments from Sanofi increased from $40,872 in 2016 to $862,320 in 2019. Similarly, Eli Lilly increased their payment from $150,707 in 2016 to $568,142 in 2019.

**Table 5:** Top ten pharmaceutical companies making the largest personal payments to the board-certified dermatologists in Japan between 2016 and 2019.

Company name	Payments by year, $				Combined total payment amounts, $ (%)	Number of payments, n (%)	Number of dermatologists receiving payments, n (%)
	2016	2017	2018	2019			
Maruho	1,499,842	1,642,352	1,821,185	1,039,835	6,003,214 (18.1)	6888 (15.8)	1515 (22.0)
Kyowa Kirin	616,397	739,856	928,888	900,078	3,185,219 (9.6)	4205 (9.7)	1158 (16.8)
Taiho pharmaceutical	438,469	780,914	911,982	1,048,121	3,179,486 (9.6)	4685 (10.8)	1476 (21.4)
Mitsubishi Tanabe pharmaceutical	581,015	692,643	748,303	578,819	2,600,780 (7.8)	3140 (7.2)	984 (14.3)
Janssen pharmaceutical	408,928	403,550	636,796	541,685	1,990,959 (6.0)	2176 (5.0)	460 (6.7)
Novartis pharma	325,323	341,905	454,269	659,795	1,781,292 (5.4)	2385 (5.5)	593 (8.6)
Sanofi	40,872	115,216	611,295	862,320	1,629,703 (4.9)	2176 (5.0)	629 (9.1)
Kaken pharmaceutical	263,546	422,248	384,932	340,024	1,410,749 (4.2)	2344 (5.4)	1,142 (16.6)
Eli Lilly Japan	150,707	282,934	368,480	568,142	1,370,262 (4.1)	1635 (3.8)	376 (5.5)
Eisai	115,518	217,579	294,964	613,234	1,241,295 (3.7)	1802 (4.1)	566 (8.2)

## Discussion

Contrary to our hypothesis, this study demonstrated that a large number but less than half (45.3%) of board-certified dermatologists received personal payments for reimbursement of lecturing, consulting, and writing from the pharmaceutical companies in Japan. The personal payments to dermatologists totaled $33,223,806 in monetary values and 43,475 in the number of payments between 2016 and 2019. Only the small number of dermatologists accepted substantial amounts of payments. Additionally, these personal payments have significantly increased during this 4-year period. There were gender differences in the patterns of payment receipt. Dermatologists with specialist certifications in dermatology-oncology and cosmetic oncology received larger personal payments from the pharmaceutical companies. To the best of our knowledge to date, this study is the first analysis assessing the financial relationships between pharmaceutical companies and all board-certified dermatologists in Japan. Although we previously reported the physician-industry financial relationships in several specialties, this study added several important insights into this issue in Japan.

Notably, we found that proportion of physicians receiving personal payments in dermatology was the lowest among previously documented specialties^4–8,28^. The proportions of physicians receiving payments ranged from 64.7% in hematology^5^ to 70.7% in oncology^7^ in Japan. Feng et al. reported 73.7% of all dermatologists received one or more payments from the healthcare industry in the United States in 2014^26^. Additionally, per-physician payments to dermatologists were also one of the lowest in Japan, followed by pediatric oncologists^4–8,28,32^. The reason for this lower payments may be explained by our limitation in data collection. We included only payments for lecturing, consulting, and writing purposes, and could not collect payments for more prevalent categories such as meals, travel and accommodations, and educations, which were widely made to dermatologists in the United States. Compensation for lecturing, consulting and writing was generally targeted at physicians with extensive clinical or research experience, typically physicians working in universities or general hospitals^6,16,33^. According to a survey by the Japanese Ministry of Health, Labor, and Welfare in 2020, 60.3% of dermatologists worked in clinics and hospitals with less than 20 inpatient beds in Japan^34^. Considering this nature, we may have underestimated the whole dermatologist-industry financial relationships in Japan.

However, we illustrated that these personal payments were increasingly made to board-certified dermatologists from the pharmaceutical companies in Japan since 2016. Behind the increasing trends in personal payments, there were increasing introduction of novel biologic drugs for atopic dermatitis and psoriasis in Japan. Our findings that the dermatologists working in the JDA-accredited facilities for use of biologics received significantly larger payments support this assumption. Remarkable progress in novel drugs for atopic dermatitis and psoriasis have been recorded worldwide. Many novel biologic drugs based on different pathways such as risankizumab (Skyrizi^®^ marketed by AbbVie was approved in March 2019)^35^, secukinumab (Cosentyx^®^ manufactured by Novartis Pharma and marketed by Maruho was approved in December 2014)^36^, brodalumab (Lumicef marketed by Kyowa Kirin was approved in July 2016)^37^, and adalimumab (Humira manufactured by AbbVie and marketed by Eisai was approved in June 2016)^38^ were introduced into psoriasis treatment. Also, dupilumab (Dupixent^®^ marketed by Sanofi) was introduced for atopic dermatitis treatment in January 2018. In Japan, most of these novel biologics for psoriasis and atopic dermatitis are available only to dermatologists working at JDA-certified facilities. It is reasonable for the pharmaceutical companies to market their products to them and ask them to give lectures other dermatologists. Due to the introduction of novel biologic drugs for atopic dermatitis, increasing trends in personal payments from healthcare industry was also observed among allergists and clinical immunologists in the United States^39^. Considering that five biologic drugs including baricitinib, delgocitinib, upadacitinib, nemolizumab, and abrocitinib additionally gained approval for atopic dermatitis after 2020, the personal payments from the pharmaceutical companies to the dermatologists will continue to increase in Japan.

Additionally, our study has added novel insights in the context of previous studies. Only a small number of dermatologists including dermatologists with specialist certifications received substantial amounts of personal payments from the pharmaceutical companies. We previously reported that the JDA clinical practice guidelines authors received from $4127 to $7043 in median annual payments. The JDA executive board members received $24,213 in median per-member payments from the pharmaceutical companies in 2016. Considering median annual personal payments were $882‒$1,046 among the board-certified dermatologists, this study provided evidence that the personal payments to influential dermatologists such as clinical practice guideline authors and society board members were considerably higher than those to general dermatologists. Unlike the physicians conducting clinical trials or developing novel therapies, considering leading physicians who are in highly ethical and authoritative positions such as professional medical society board members and clinical practice guideline authors have considerable impact on other physicians’ clinical practice and patient care, these physicians must be independent and minimize their financial relationships with the pharmaceutical companies^40–42^. Otherwise, these leading physicians at least must be transparent about their financial relationships with the pharmaceutical companies, though most of the financial relationships between the leading dermatologists and the pharmaceutical companies were undisclosed and underdeclared by the dermatologists in Japan^16^.

There were large gaps in personal payments between male and female dermatologists in Japan, which was consistent with our previous study assessing the financial conflicts of interest among the JDA clinical practice guidelines authors^16^. The gender differences in industry payments are well-described in the United States as well, as fewer female physicians received lower personal payments from the pharmaceutical and medical devices manufacturers than male physicians in the United States^43–49^. Many factors would have contributed to the lower personal payments from the pharmaceutical companies to female physicians in Japan. First, Female physicians tended to have lower motivation for negotiating their payments and accepted lower earnings for non-monetary benefits such as flexibility of work time and location. Therefore, female dermatologists might not have negotiated the lower personal payments and might have declined the lecturing events sponsored by the pharmaceutical companies in Japan. Other possible reason is the lower representation of female physicians in Japanese medical society. Harada et al. reported that only 5.7% (20 out of 351) of all Japanese editors-in-chiefs of medical academic journals were female^50^. Of 296 dermatology clinical practice guideline authors, only 49 equal to 16.6% were female in Japan^16^. Thus, the lower personal payments to the dermatologists might be due to the lower presentations in academic and research positions in Japan. Future study should examine the lower personal payments to the female dermatologists from the pharmaceutical companies adjusting other covariables.

This study included several limitations. First, as the transparency guidance was a voluntary self-disclosure of the payments by the pharmaceutical companies, although unethical, the pharmaceutical companies may not have accurately disclosed and hidden the payment data. Additionally, though physicians can correct the payment data by personal contacts with the companies, there was no official dispute and correction process in the current self-disclosure in Japan. Thus, there were possibilities in inaccuracies of payment data disclosed by the companies. Second, despite we repeatedly cross-checking the collected data for any errors by two or more persons, the inclusion of errors in the collected data by our study team could not be ruled out. Third, as the payment data concerning various categories including meals, educational activities, transportation and accommodations were not disclosed with individual name of recipients by the pharmaceutical companies in Japan^3^, this study would have underreported the prevalence and magnitude of financial relationships between the dermatologists and pharmaceutical companies in Japan. Fourth, there were possible confounding factors influencing the personal payments to the dermatologists other than dermatologists’ gender and specialist certification which were not included in the database of the JDA board-certified dermatologists.

In conclusion, this study found that 45.3% of Japanese board-certified dermatologists received lower personal payments for the reimbursement of lecturing, consulting, and writing than those to other specialists between 2016 and 2019. Only a small portion of dermatologists, such as those with specialist certification and male sex, received vast majority of payments from the pharmaceutical companies. Furthermore, these personal payments were increasingly more prevalent and greater over the 4 years during the study period.

## Data Availability

The datasets generated during and/or analyzed during the current study are available from the corresponding author on reasonable request.
